# Evidence for the evolution of native plant response to mycorrhizal fungi in post‐agricultural grasslands

**DOI:** 10.1002/ece3.9097

**Published:** 2022-07-11

**Authors:** Camille S. Delavaux, James D. Bever

**Affiliations:** ^1^ Department of Ecology and Evolutionary Biology The University of Kansas Lawrence Kansas USA; ^2^ Kansas Biological Survey The University of Kansas Lawrence Kansas USA; ^3^ Department of Environmental Systems Science ETH Zurich Zurich Switzerland

**Keywords:** anthropogenic change, Glomeromycota, grasslands, greenhouse experiment, growth response, mycorrhizal response

## Abstract

Plant–microbe interactions play an important role in structuring plant communities. Arbuscular mycorrhizal fungi (AMF) are particularly important. Nonetheless, increasing anthropogenic disturbance will lead to novel plant–AMF interactions, altering longstanding co‐evolutionary trajectories between plants and their associated AMF. Although emerging work shows that plant–AMF response can evolve over relatively short time scales due to anthropogenic change, little work has evaluated how plant AMF response *specificity* may evolve due to novel plant–mycorrhizal interactions. Here, we examine changes in plant–AMF interactions in novel grassland systems by comparing the mycorrhizal response of plant populations from unplowed native prairies with populations from post‐agricultural grasslands to inoculation with both native prairie AMF and non‐native novel AMF. Across four plant species, we find support for evolution of differential responses to mycorrhizal inocula types, that is, mycorrhizal response specificity, consistent with expectations of local adaptation, with plants from native populations responding most to native AMF and plants from post‐agricultural populations responding most to non‐native AMF. We also find evidence of evolution of mycorrhizal response in two of the four plant species, as overall responsiveness to AMF changed from native to post‐agricultural populations. Finally, across all four plant species, roots from native prairie populations had lower levels of mycorrhizal colonization than those of post‐agricultural populations. Our results report on one of the first multispecies assessment of local adaptation to AMF. The consistency of the responses in our experiment among four species provides evidence that anthropogenic disturbance may have unintended impacts on native plant species' association with AMF, causing evolutionary change in the benefit native plant species gain from native symbioses.

## Graphical abstract

Here, we examine changes in plant‐AMF interactions in novel grassland systems by comparing the mycorrhizal response of plant populations from unplowed native prairies with populations from post‐agricultural grasslands to inoculation with both native prairie AMF and non‐native novel AMF. Across four plant species, we find support for evolution of differential responses to mycorrhizal inocula types consistent with expectations of local adaptation, with plants from native populations responding most to native AMF and plants from post‐agricultural populations responding most to non‐native AMF. Our results highlight that widespread anthropogenic disturbance can have unintended impacts on native plant species’ association with AMF, causing evolutionary change in the benefit native plant species gain from native symbioses.

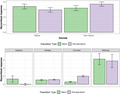

## 1 INTRODUCTION

There has been a growing understanding of the vital links between plant communities and their soil microbiome (Barberán et al., 2015; Bever et al., 2010; Delgado‐Baquerizo et al., 2016), with both mutualistic and pathogenic microbes implicated in the maintenance of plant diversity. Evidence to date shows that plant pathogens structure plant communities and maintain plant community diversity (Bever et al., 2015; Crawford et al., 2019; Eppinga et al., 2018; Mordecai, 2011; van der Heijden et al., 2008; Van der Putten et al., 2013). A major group of mutualists, mycorrhizal fungi, are also important in determining plant community structure (Bever, 2002; Mangan et al., 2010; van der Heijden et al., 1998; Vogelsang et al., 2006). These symbionts play an important role in mediating plant succession (Janos, 1980; Koziol & Bever, 2015, 2019) and in the establishment and distribution of plant species worldwide (Delavaux et al., 2019, 2021).

Plant interactions with arbuscular mycorrhizal fungi (AMF) are particularly important in native systems, with both partners coevolving over time. There is strong evidence of coevolution of the relationship between plants and their mycorrhizal fungi (Brundrett, 2002). Indeed, AMF are hypothesized to have played a major role in land colonization by plants, serving as root‐like structures to aquatic plants (Redecker et al., 2000). Evidence shows that the arbuscular mycorrhizal state is the ancestral state, with later plant groups evolving ectomycorrhizal or non‐mycorrhizal relationships (Brundrett, 2002; Maherali et al., 2016). Further, this evolutionary history has been shown to be a dominant driver of mycorrhizal response. A recent meta‐analysis (Hoeksema et al., 2018) showed that evolutionary history is a strong driver of plant response to mycorrhizal fungi, and is more important in determining the interaction outcome than other traditionally studied environmental moderators. Selection and breeding in agriculture also offer evidence for more recent evolutionary influence on the plant–AMF interaction, often leading to lower responsiveness to AMF (Koziol et al., 2012; Martín‐Robles et al., 2018; Turrini et al., 2016).

Increasingly, anthropogenic disturbance, including species introductions and range shifts, will lead to novel plant–microbe interactions, interfering with this longstanding co‐evolutionary trajectory (Dickie et al., 2017; Pringle et al., 2009). Evidence suggests that these changes may result in relatively rapid evolution, with AMF responsiveness evolving over a few decades. Invasion offers an important perspective into formation of novel plant–mycorrhizal relationships, with invasive plants often showing reduced response to AMF (Cheeke et al., 2019; Pringle et al., 2009; Seifert et al., 2009), or directly reducing AMF abundance (Callaway et al., 2008; Crawford et al., 2019; Stinson et al., 2006; Vogelsang & Bever, 2009), but see (Bunn et al., 2015). Seifert et al. (2009) found that St. John's wort showed reduced responsiveness to AMF during invasion of North America compared to native source populations. As St. John's wort is abundant in anthropogenically disturbed areas of North America, this study suggests that evolution of mycorrhizal response may occur due to anthropogenic disturbance. Nonetheless, it is unknown whether the specificity of plant response to AM fungal composition may also evolve rapidly in general and specifically in response to anthropogenic disturbance.

The tallgrass prairie system in the Midwestern US is an ideal system in which to ask questions related to evolution of mycorrhizal response and specificity, with a long history of research into the ecology of plant–mycorrhizal relationships. Work in tallgrass prairies has shown that plant–mycorrhizal interactions sustain native plant diversity, with late successional prairie dominants showing high responsiveness to AMF and fungal composition (Cheeke et al., 2019; Koziol & Bever, 2015, 2016, 2019; Vogelsang et al., 2006; Wilson & Hartnett, 1998). Further, as most prairie systems have been altered by human activity (Samson et al., 2004), novel plant–microbial interactions are dominant in these heavily degraded systems. Specifically, anthropogenic disturbance of prairies has been shown to degrade AMF communities (House & Bever, 2018), and reintroduction of native AMF into these settings increases the establishment success and growth of late successional prairie plant species (Koziol & Bever, 2017, 2019; Lubin et al., 2019; Middleton et al., 2015). However, a subset of native prairie plant species has been more successful in colonizing in post‐agricultural sites, often becoming more abundant than they were in native prairies. These early successional native prairie plant species have been shown to be less dependent on AMF than late successional prairie plant species (Bauer et al., 2018; Koziol & Bever, 2015) and their response is less sensitive to AMF species identity (Cheeke et al., 2019; Koziol & Bever, 2016). However, whether these early successional species differentially respond to native AMF and whether they have evolved in their relationship with AMF during colonization of post‐agricultural sites has not been explored.

Here, we investigate the evolution of plant mycorrhizal response and the specificity of this response across tallgrass prairies in Eastern Kansas, USA. We first test for differences in mycorrhizal responsiveness between two plant population types, native and post‐agricultural, across four native plant species. We then test for differences in specificity of mycorrhizal response by comparing the response of these population types to native prairie AMF inocula with the response to novel non‐native AMF inocula. Local adaptation predicts that populations from undisturbed prairies will benefit most from native prairie AMF, while populations from disturbed prairies will benefit most from novel AMF (Kawecki & Ebert, 2004; Núñez‐Farfán & Schlichting, 2001; Williams, 2018). This work clarifies how populations of the same native plant species in native and post‐agricultural prairies differ in their growth response to mycorrhizal fungi generally (mycorrhizal response) and to inocula origin specifically (mycorrhizal response specificity), informing potential evolutionary consequences of novel interactions resulting from anthropogenic change.

## 2 MATERIALS AND METHODS

### 2.1 Site description

To test differences in mycorrhizal response between native and post‐agricultural plant populations, we selected eight prairie sites across Kansas, USA. Four sites were classified as native, representing native prairies sites without human disturbance. The four remaining sites were post‐agricultural sites which were abandoned agricultural fields (between 20 and 50 years) and represent novel, disturbed sites. The native sites included two sites at the University of Kansas Field Station (Rockefeller Prairie, 39.0°N, 95.2°W, and Dogleg Prairie, 30.1°N, 95.2°W), Prairie Nature Park Prairie (38.9°N, 95.2°W), and Kill Creek Prairie (38.9°N, 95. 0°W). The post‐agricultural sites included a site at The Land Institute in Lawrence, KS (39.0°N, 95.2°W), two sites of the University of Kansas Field station (Welda Prairie, 38.2°N, 95.3°W, and Plot 4010, 39.1 °N, 95.2 °W) and the Rock Chalk Park walking trails (39.93°N, 95.33°W).

### 2.2 Seed collections

Seeds were hand collected during September and October 2018. Plant species were chosen to represent those species that are native tallgrass prairie, but easily colonize post‐agricultural sites as well, representing early successional species. The following species were collected from each site when possible for this study: *Apocynum cannabinum* (Dogbane)*, Vernonia fasciculate* (Ironweed)*, Asclepias syriaca* (Milkweed)*,* and *Solidago canadensis* (Solidago). Seeds were air dried, hand separated and stored at 4°C in a walk‐in fridge.

### 2.3 Mycorrhizal inocula

Two types of mycorrhizal inocula mixtures were used in this study to test the differential response of each plant population type to each type of mycorrhizal fungi. To represent mycorrhizal fungal species found in native prairie sites, we used a mixture of 11 AMF species cultured from nearby prairie remnants. To represent novel mycorrhizal fungal species as might be found in disturbed post‐agricultural sites (House & Bever, 2018), we used 10 AMF species from the International Culture Collection of Vesicular Arbuscular Mycorrhizal Fungi (INVAM, West Virginia University; Table S1). Note that these AMF are not from post‐agricultural sites, but represent novel non‐native AMF. Nonetheless, our goal was to compare native to novel plant–mycorrhizal relationships; therefore, use of novel, foreign AMF was appropriate for our study. Furthermore, we chose species that we knew to be present in post‐agricultural grasslands of the prairie region of the United States based on identification of spores and environmental sequencing (House & Bever, 2018; Table S2). Species were chosen for each inocula mixture to represent a broad and comparable phylogenetic spread across the AMF clade. Each AMF species used in the mixtures was grown as single species culture in the same greenhouse, soil, and with the same plant hosts during the previous summer. Native AMF inoculum was generated on a mixture of prairie plant species, while novel AMF inoculum was generated on Sorghum. Equal proportions of AMF species were mixed to create the two inocula communities of distinct origins used in the experiment.

### 2.4 Greenhouse assay

In Spring 2019, seeds were cleaned and planted in sterile potting soil and cold stratified for 6 weeks. The planted seeds were then placed in a greenhouse to germinate for 2 weeks. Three seedlings from a given individual were taken and planted in each of three soil treatments: sterile, native prairie fungi, or non‐native fungi, with five replicates (individuals) for each treatment per plant species per site, for a total of 28 test populations (Table S3). AMF inoculum was added to the plant root zone as a mixture of rhizosphere soil, including soil and roots. Plants were then grown for 13 weeks in the greenhouse. At harvest, we measured plant height and weighed dried aboveground and belowground biomass. Biomass was dried at 70°C until biomass reached a constant weight. All roots (belowground biomass) were hand cleaned to remove soil and small pebbles.

### 2.5 Root architecture and colonization

We conducted root architecture (root length) analysis on entire root systems of plants in the sterile treatment. Root architecture in the sterile treatment has been found to correlate with mycorrhizal response (Koziol & Bever, 2015; Seifert et al., 2009); smaller root systems tend to be more responsive to mycorrhizal fungi, whereas larger ones tend to be less responsive. Root systems were quantified using WinRhizo (Regent Instruments Inc.).

We also inspected root colonization in a subset of study plants to confirm that our sterile controls did not have AMF colonization and that the AMF treatments showed AMF colonization. Root colonization was assayed using Trypan Blue staining and subsequent microscope inspection (Giovanetti & Mosse, 1980). Stained roots were mounted on slides and were analyzed using 25 vertical transects for colonization as well as for presence of arbuscules, vesicles, coils, and hyphae (Table S4).

### 2.6 Statistical analyses

We tested for differences in mycorrhizal colonization between AMF inoculation treatments and differences in colonization across the population types and plant species by using a generalized linear mixed model (GLMM) predicting colonized to non‐colonized counts of mycorrhizal occurrence as a combined response variable, specifying the binomial family with a logit link. We tested for consistent differences between sterile and inoculated treatments treating AMF inocula as a fixed effect and the interaction between plant species and population type as a random effect. Because the AMF colonization level in the sterile treatment was near zero, in a second analysis, we omitted the sterile treatment and analyzed the inoculated plants to test whether the population types differed in overall infection. We tested for consistent differences between population types across the four plant species by treating population type, plant species, AMF inocula, and the interaction of plant population type by AMF inocula as fixed effects and the interaction of population type within plant species and of this term with AMF inocula as random effects. To analyze the difference in measured root length between plant species, we used a linear model predicting specific root length (total root length ÷ root biomass) predicted from plant species, and included the random effect of plant species interacting with plant population type. We reran this model to test for the interaction between plant species and population type. These statistical analyses were carried out using R v. 3.6.0 (RC Team, 2019) using the lme4 (Bates et al., 2015), lmerTest (Kunzetsova et al., 2017), and tidyverse (Wickham et al., 2019), with plotting using the lsmeans (Lenth, 2016), gridExtra (Auguie et al., 2017), and ggplot2 packages (Wickham, 2009).

To test mycorrhizal growth response (biomass), we used generalized linear mixed effect (GLMM) models to predict either aboveground or belowground biomass. These models test whether the two population types differed consistently in growth response to inoculation across the eight replicate populations of each of the four plant species. We first conducted these analyses on all data across the four plant species, and when the interactions with plant species were significant, we tested each plant species individually. For these analyses, no single transformation adequately homogenized the variation between treatments. We therefore took rank transformed the above‐ or below‐ground biomass within plant species as the best available option for our response variable. Our analyses thus corresponded to tests of medians of the distributions of biomass rather than means (Conover & Iman, 1981).

To predict this rank transformed biomass, we used treatment, species, population type, and initial height at planting as our independent predictor variables. Plant height of seedlings after transplanting was included as it can partly account for potential maternal effects such as seed size differences. We also included the random effects of treatment nested within species nested within population replicate nested within population type, plant species nested within plant population replicate nested within plant population type, and plant species replicate (in greenhouse experiment) nested within plant species nested within plant population replicate nested within plant population type (native or post‐agricultural) to account for non‐independence of samples. AMF inocula effects and all interactions with AMF inocula were decomposed into two orthogonal a priori contrasts that separately tested the average growth response to AMF (AMF vs. sterile) and the differences in response to the two sources of AMF (native vs. non‐native). Consistent differences between populations from native versus disturbed locations across all populations of all plant species were detected as significant population type effects in the mixed model. Consistent differences in population type interactions with mycorrhizal fungi were detected within the population type by AMF inocula interaction, which was broken into the overall mycorrhizal growth response (population type by sterile vs. AMF) and specificity of mycorrhizal growth response (population type by AMF type, native vs. non‐native origin). These GLMMs were run in SAS (SAS Institute, 2012).

As the rank transformation did not perfectly satisfy parametric assumptions, we used permutation approaches to assess the robustness of inference from the analyses of ranks. We conducted 1000 permutations to construct a *p*‐value based on the distribution of the resulting estimates; when estimates were not available we used *p*‐values instead. To do this, we resampled the data, grouping the data by plant species, reassigning the joint value of mycorrhizal treatment, population type and population replicate in R v. 3.6.0 (RC Team, 2019) using the tidyverse package (Wickham et al., 2019). We then reran the previously described GLMMs with these 1000 datasets in SAS. We then calculated the new *p*‐value based on the actual distribution of permutations in R. The plots presented here used the output from these biomass models to calculate mycorrhizal (growth) response (MGR), using the following formula: (Biomass_AMF_‐Biomass_Sterile_) ÷ Biomass_Sterile_. Mycorrhizal growth response represents the relative change in biomass due to the additional of mycorrhizal inocula.

Finally, we investigated correlations between MGR and specific root length (SRL) as well as between MGR and colonization using linear models. To investigate correlations between MGR and SRL, we used the interaction of SRL and plant species to predict MGR, with the random effect of plant species interacting with plant population type. To investigate correlations between MGR and colonization, we used MGR as the response variable predicted by the interactions of logit colonization proportion (colonization) and plant population type and of colonization and plant species, with the random effect of plant species interacting with plant population type. All model results are summarized in Table S5.

## 3 RESULTS

### 3.1 Mycorrhizal colonization

**FIGURE 1:**
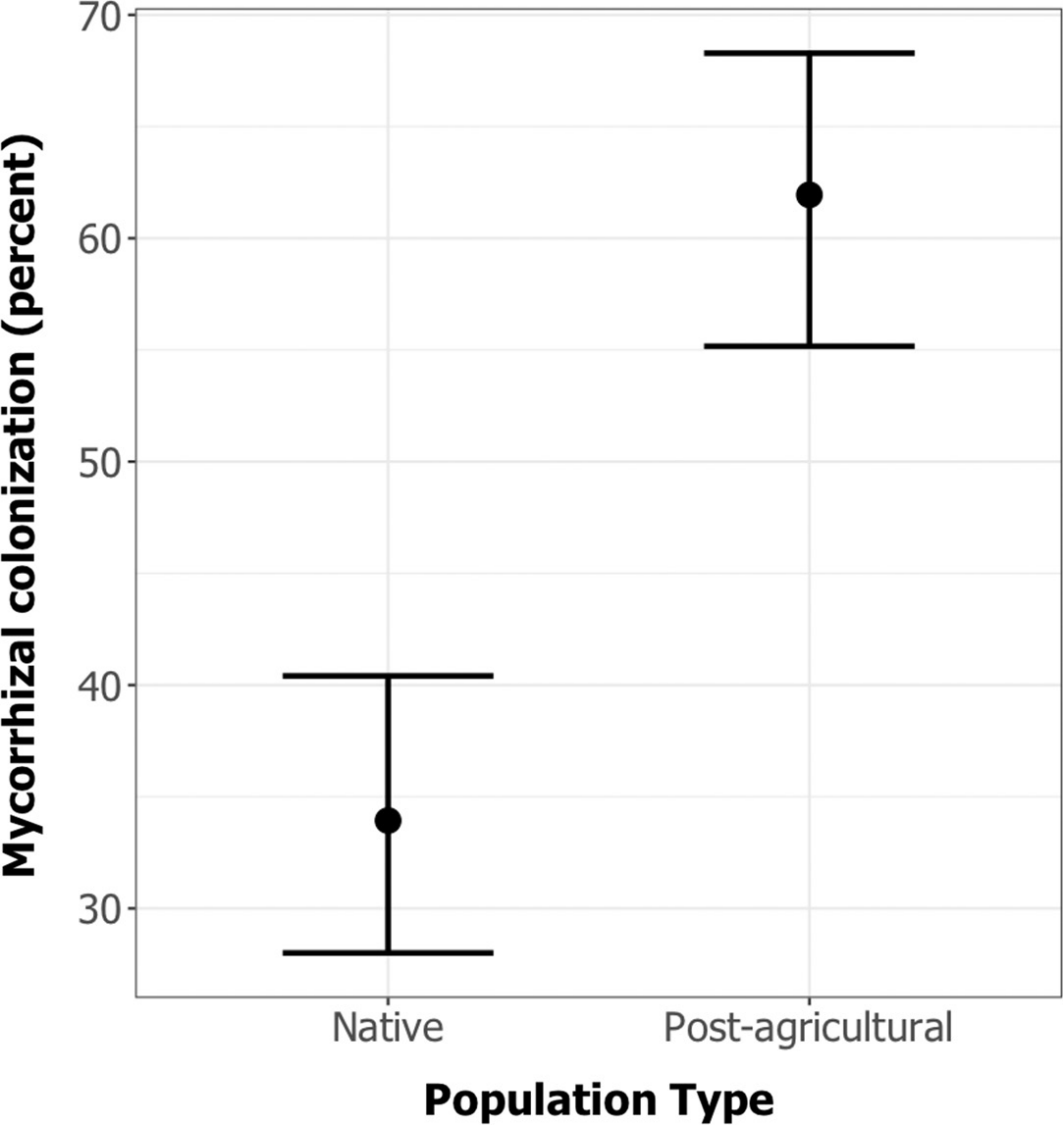
Mycorrhizal colonization differences across study species. GLM shows that there is significantly greater arbuscular mycorrhizal colonization in post‐agricultural compared to native populations (*F*_1,3_ = 16.24, *p* = .002)

We found that the mycorrhizal treatments were successful, with mycorrhizal treatments showing significantly higher colonization than sterile treatments (*F*_1,34_ = 34.07, *p* < .001), with very few instances of mycorrhizas were encountered in the sterile treatment (Figure S1; mean of 0.25 occurrences per 25 root intersections). Across all plant species, we found that post‐agricultural populations showed greater colonization than native populations (Figure 1, *F*_1,2_ = 16.24, *p* = .002), but find no plant population type by mycorrhizal inocula type interaction (*F*_1,8_ = 1.9603, *p* = .16).

### 3.2 Specific root length

We did not find that SRL varied consistently with plant population type across the four plant species (*F*_3,84_ = 0.077, *p* = .97). Comparing plant species, we found a marginally significant plant species effect with Dogbane showing longer SRL compared Ironweed (Figure S2; *F*_3,4.56_ = 3.935, *p* = .1).

### 3.3 Mycorrhizal growth response

Overall, plants grew significantly larger with AMF than in the sterile treatment, both in terms of aboveground (rank: *p* < .0001, *F*_1,175_ = 312.98; permutation: *p* < .0001) and belowground (rank: *p* < .0001, *F*_1,178_ = 201.16; permutation: *p* < .0001) biomass. When analyzing individual plant species, each plant species was mycorrhizally responsive and showed greater growth in AMF versus sterile treatments, for aboveground biomass (rank: Dogbane: *F*_1, 31.6_ = 6.30, *p* = .018; Ironweed: *F*_1,9.28_ = 129.5, *p* < .0001; Milkweed: *F*_1,34.9_ = 133.53, *p* < .0001; Solidago: *F*_1,9.8_ = 192.59, *p* < .0001; permutation: Dogbane: *p* = .019; Ironweed: *p* < .0001; Milkweed: *p* < .0001; Solidago: *p* < .0001), while almost all, with the one exception of Dogbane, was mycorrhizally responsive for belowground biomass (rank: Ironweed: *F*_1,83_ = 61.58, *p* < .0001; Milkweed: *F*_1,5.23_ = 129.88, *p* < .0001; Solidago: *F*_1,9.8_ = 126.36, *p* < .0001; permutation: Dogbane: *p* = .085; Ironweed: *p* < .0001; Milkweed: *p* < .0001; Solidago: *p* < .0001). We found a significant difference between the two types of AMF inocula in belowground biomass, with non‐native novel AMF resulting in a higher growth increase than native AMF (rank: *F*_1,180_ = 5.15, *p* = .024; permutation: *p* = .056).

### 3.4 Population differences in mycorrhizal growth response

**FIGURE 2:**
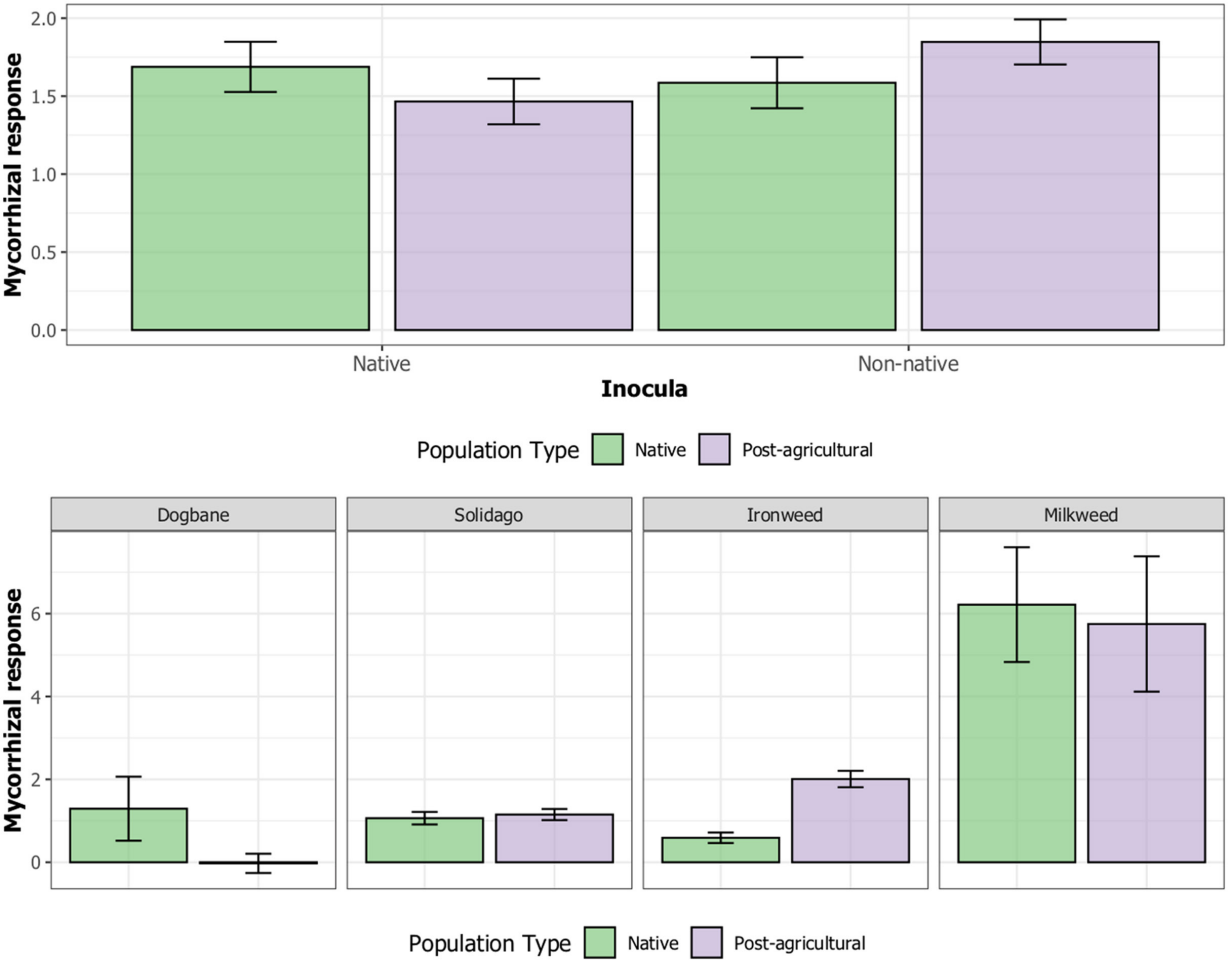
Evidence for evolution of mycorrhizal response. Across the four species included in the study, native plant populations showed the greatest mycorrhizal response to native AMF inocula, while post‐agricultural plant populations showed the greatest mycorrhizal response to non‐native AMF inocula (A, rank: *F*_1,175_ = 4.87, *p* = .03; permutation: P = 0.08). Belowground, within species, for dogbane, native plant populations are more mycorrhizally responsive than post‐agricultural plant populations (B, rank: *F*_1,31.6_ = 3.53, *p* = .07, permutation *p* = .058), while for ironweed, post‐agricultural populations are more responsive than native plant populations (rank: *F*_1,83_ = 6.56, *p* = .01; permutation: P = 0.036)

We found consistent differences between the two plant population types in specificity of response to the two types of AMF inocula. Across all four plant species, we found post‐agricultural plant populations generally grew best with to non‐native AMF, while native plant populations grew best with native AMF inocula, with a significant interaction between origin of AMF inocula and plant population for aboveground biomass (Figure 2a; rank: *F*_1,175_ = 4.87, *p* = .03; permutation: *p* = .08).

We also found differences between population types in overall mycorrhizal responsiveness (regardless of AMF type), but this effect varied significantly with plant species, with a significant three‐way interaction between AMF versus sterile by plant population type by plant species for belowground biomass (Figure 2b, rank: *F*_3,177_ = 4.46, *p* = .0048; permutation: *p* < .0001). Specifically, for Dogbane, native plant populations were more mycorrhizally responsive than post‐agricultural plant populations (rank: *F*_1,31.6_ = 3.53, *p* = .07, permutation *p* = .058), while for Ironweed, post‐agricultural populations were more responsive than native plant populations (rank: *F*_1,83_ = 6.56, *p* = .01; permutation: *p* = .036). This pattern was found aboveground as well, but was weaker (rank: *F*_3,174_ = 2.56, *p* = .06; permutation: *p* < .0001).

### 3.5 Mycorrhizal growth response, specific root length and mycorrhizal colonization

**FIGURE 3:**
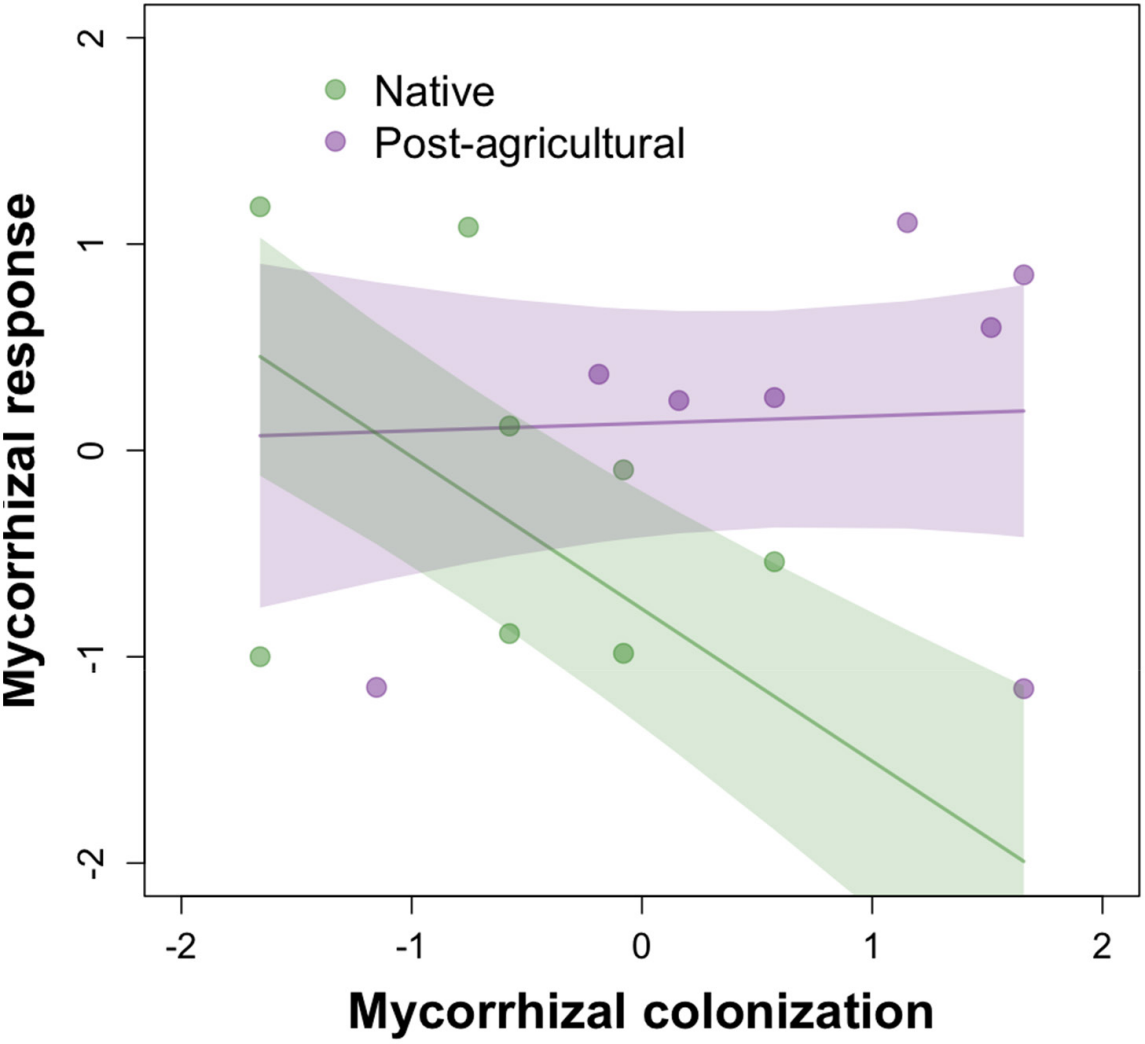
Population type differences in relationship between mycorrhizal colonization and response. The relationship between logit colonization proportion and mycorrhizal growth response depends on population type, with native populations showing a more negative relationship (*p* = .01)

We did not find an overall correlation between SRL and mycorrhizal growth response (MGR; *F*_1,17_ = 0.535, *p* = .47). We found that this relationship varied depending on plant species, and found an interaction between plant species and SRL in predicting mycorrhizal growth response (*F*_3,17_ = 5.123, *p* = .01). Specifically, Solidago and Ironweed showed a positive correlation between MGR and SRL, while Milkweed showed a negative correlation. Further, we found a significant interaction between colonization and plant population type in predicting MGR (Figure 3, *F*_1, 5.94_ = 14.395, *p* < .01), with a more negative correlation in native than post‐agricultural plant populations. We also found a significant interaction between colonization and plant species (Figure S3, *F*_3,4.68_ = 8.98, *p* = .02), with Ironweed showing a positive correlation, while other plant species showed a negative correlation.

## 4 DISCUSSION

Here, we found evidence of evolution of plant–mycorrhizal interactions in anthropogenically disturbed, post‐agricultural grasslands. Across four native plant species, we found that post‐agricultural plant populations had higher mycorrhizal colonization than populations from undisturbed grasslands. We also found that populations from undisturbed grasslands generally showed greater mycorrhizal response to native AMF, while populations from post‐agricultural grasslands showed greater response to novel, non‐native AMF. These two results were consistent across the four plant species tested in our study, suggesting that the plant–AMF interaction evolved in response to anthropogenic disturbance in predictable directions. We also found evidence that overall AMF responsiveness evolved in post‐agricultural populations in two of the plant species. These findings suggest that overall mycorrhizal response and mycorrhizal response to specific AMF can evolve within a relatively rapid timeframe, highlighting potential evolutionary consequences of anthropogenic disturbance on plant–mycorrhizal interactions.

We found novel evidence for the evolution of mycorrhizal response specificity. Plants from native populations responded more positively to native AMF, while plants from populations colonizing disturbed, post‐agricultural, areas responded more positively to non‐native AMF. Although this result was only marginally statistically significant, it was in the direction predicted from a priori expectations of plant–AMF co‐adaptation. Moreover, this shift in responsiveness was accompanied by a statistically robust shift in levels of mycorrhizal colonization. Specifically, we find populations colonizing disturbed land (post‐agricultural populations) consistently exhibited greater mycorrhizal colonization rates regardless of the inocula source compared to populations from unplowed, native prairies. In addition, we found that native plant populations showed a negative relationship between AMF colonization and growth response to AMF, while post‐agricultural populations did not. That native plants were less colonized overall and showed greatest mycorrhizal response with lower colonization suggests that native populations are more selective in their mycorrhizal associations, perhaps because they benefit most from native AMF. In contrast, post‐agricultural plant populations had higher colonization rates and showed no relationship between mycorrhizal colonization and response, suggesting that these plants are more permissive of AMF infection.

Our results provide evidence for the evolution of overall AMF response during colonization of disturbed land in eastern Kansas, though the direction of this effect varied between two species. We found that Dogbane mycorrhizal responsiveness decreased, while Ironweed mycorrhizal responsiveness increased in post‐agricultural plant populations. Anthropogenic disturbance results in strong degradation of the AMF community (House et al., 2016) and in benefit to native prairie plant species (Koziol & Bever, 2019). Evolution of decreased mycorrhizal response in anthropogenically disturbed land is consistent with expectations based on this loss of AMF function. This is also consistent with previous work showing evolution of reduced response to AMF of invasive plants which dominate in disturbed lands of North America (Seifert et al., 2009), and loss of response during selection for yield in disturbed agricultural systems (Koziol et al., 2012; Martín‐Robles et al., 2018; Turrini et al., 2016).

The increased mycorrhizal growth response in Ironweed post‐agricultural populations is counter to our a priori expectation. The variation in response between these plant species suggests that factors other than the degradation of AMF may be important in determining the direction of evolution of mycorrhizal response. For example, the absence of competition from later successional plant species in post‐agricultural lands may generate selection for more late successional traits, such as high responsiveness to AMF (Bauer et al., 2018), in mid‐successional Ironweed. Alternatively, tradeoffs with pathogen defense, a non‐nutritional benefit of AMF (Delavaux et al., 2017) could alter simple expectations. There is evidence for the importance of mycorrhizal induced resistance by native AMF in grassland systems through reduced effects of herbivory (Middleton et al., 2015) and fungal pathogens (Sikes et al., 2009). Further research will be needed to experimentally test relative pathogen impacts on these plant populations and respective AMF communities within these plant species.

Although mycorrhizal fungi are known to be important in tallgrass prairie systems, the early successional plant species such as those used in this study have generally been shown to be less responsive (Bauer et al., 2018; Koziol & Bever, 2015) and less sensitive to AMF identity (Cheeke et al., 2019; Koziol & Bever, 2016) than late successional plant species. However, we found that these early successional species were responsive to AMF, consistent with Reynolds et al. (2020) and that responsiveness can vary with the population sources and with AMF identity.

While our study provides the first evidence of relatively rapid evolution of mycorrhizal response specificity in response to anthropogenic disturbance, we must qualify our confidence in attribution of the observed shifts in the mycorrhizal interactions from native to post‐agricultural populations as an evolutionary shift. As we used field collected seeds, it is possible that environmental differences in seed origin contributed to maternal differences, such as seed provisioning, and that this influenced our observed responses. We note that we attempted to minimize this possibility, by including collections from independent mother plants as our replicates within each of multiple independent populations within each plant species, by germinating all seed in a common environment, and by including initial height of seedlings at planting as a partial control of potential differences in maternal provisioning. Nevertheless, further work reducing the potential for confounding maternal effects and identifying the genetic basis of the shifts in plant responses to mycorrhizal fungi is needed to confirm the evolutionary nature of the observed phenotypic shifts. While we observed these phenotypic shifts within sites that have been abandoned for between one to a few decades, we note that we cannot be definitive on the timescale of the potential evolutionary response. Because we do not know the precise origin and evolutionary trajectory of these plant populations, we acknowledge that they may have been evolving in disturbed locations before colonizing the post‐agricultural sites sampled in this study.

We show for the first time that evolution of mycorrhizal response specificity is possible over relatively short timescales following disturbance. The native plant species targeted in this study showed a shift in overall growth response based on both plant population type and AMF inocula type, with native plant populations more responsive to native AMF inocula and post‐agricultural populations more responsive to non‐native AMF inocula. Our results highlight the sensitivity of native plant–mycorrhizal associations to human disturbance. On a practical level, our work supports the importance of plant source (population type) and mycorrhizal inocula origin for reestablishing grasslands. More generally, continuing anthropogenic disturbance has the potential to alter the longstanding co‐evolution trajectory of plants and their associated mycorrhizal fungi. Here, we find that this anthropogenic change leads to shifts in the functionality of this plant–mycorrhizal relationship, with consequences for our understanding of plant–AMF co‐evolution and strategies to recreate native ecosystems.

## AUTHOR CONTRIBUTIONS

**Camille Suzanne Delavaux:** Conceptualization (lead); data curation (lead); formal analysis (lead); investigation (lead); project administration (lead); resources (supporting); software (equal); writing – original draft (lead); writing – review and editing (lead). **James D. Bever:** Conceptualization (supporting); formal analysis (equal); funding acquisition (lead); project administration (supporting); supervision (supporting); writing – original draft (supporting); writing – review and editing (supporting).

## CONFLICT OF INTEREST

The authors declare no conflict of interest.

## Supporting information


**Appendix S1** Supporting Information

## Data Availability

Data and code are available from github at https://github.com/c383d893/Evolution‐of‐Mycorrhizal‐Response with DOI: 10.5281/zenodo.4521299.
