# Supplementary material for: Evidence for the evolution of native plant response to mycorrhizal fungi in post‐agricultural grasslands
Source: Ecol Evol. 2022 Jul 11;12(7):e9097. doi: 10.1002/ece3.9097 (PMC9273508; doi:10.1002/ece3.9097)

**Supporting Information**

**Title: Evidence for the evolution of native plant response to mycorrhizal fungi in post-agricultural grasslands**

**Table S1. Mycorrhizal inocula used in the study.** Eleven cultures were used for native prairie AMF (all from native KS tallgrass prairies); ten cultures were used for non-native (INVAM) AMF (all not from native KS tallgrass prairies).

| **Native AMF** | | **Non-native AMF** | | |
| --- | --- | --- | --- | --- |
| Name | Likely species | Name | Species | Origin |
| Sanders Mound B | *Ambispora sp.* | NC119-24 | *Gigaspora gigantea* | *NC, USA* |
| Sanders Mound F | *Rhizophagus sp.* | IN212 | *Racocetra fulgida* | *IN, USA* |
| Trap 18C | *Rhizophagus sp.* | BR208A | *Cetraspora pellucida* | *Brazil* |
| Scut 305-3 Insta B | *Cetraspora pellucida* | FL327C | *Archaeospora trappei* | *FL, USA* |
| Trap 18G | *Cetraspora pellucida* | IA122 | *Paraglomus occultum* | *IA, USA* |
| Sanders Mound 0 | undetermined | IN218 | *Ambispora leptoticha* | *IN, USA* |
| 508-2 | *Gigaspora gigantea* | MT106 | *Ambispora gerdemannii* | *MT, USA* |
| 514-1 | *Gigaspora gigantea* | BR851 | *Rhizophagus clarus* | *Brazil* |
| 507-2 | *Funniliformis mosseae* | FL328 | *Septoglomus constrictum* | *FL, USA* |
| 513-2 | *Glomus mortonii* | AZ242 | *Funneliformus mosseae* | *AZ, USA* |
| Rock GZ 187 E. inf | *Claroidoglomus claroideum* |  |  |  |

**Table S2. Non-native AMF species used in study identified in post-agricultural tallgrass prairie ecosystems.** Several species used in our study as non-native novel AMF representing taxa likely to occur in post-agricultural tallgrass prairies are indeed found in disturbed Kansas tallgrass prairies as well as disturbed Midwestern tallgrass prairies.

**Table S3. Greenhouse experimental design.** Greenhouse experimental design to test mycorrhizal response between native and post-agricultural plant population types to native and non-native mycorrhizal inocula. Numbers represent number of replicates per species per site. Each replicate individual was planted in each treatment (sterile, native prairie fungi, or non-native fungi).

| **Site** | ***Apocynum cannabinum* (Dogbane)** | ***Solidago Canadensis***  **(Solidago)** | ***Vernonia faciculata* (Ironweed)** | ***Asclepias sp.* (Milkweed)** |
| --- | --- | --- | --- | --- |
| **Native Plant Populations** | | | | |
| Rockefeller | 5 | 5 | 5 | 5 |
| Dogleg | 5 | 5 | 5 | 5 |
| Kill Creek Prairie | 5 | 5 | 5 | 5 |
| Prairie Nature Park | 0 | 5 | 0 | 0 |
| **Post-agricultural Plant Populations** | | | | |
| Welda | 5 | 5 | 5 | 5 |
| Plot 4010 | 5 | 5 | 5 | 0 |
| Land Institute Property | 5 | 5 | 5 | 5 |
| Rock Chalk Park | 5 | 5 | 5 | 0 |

**Table S4. Mycorrhizal assessment results.** Mycorrhizal identification in a subset of samples using the gridline intersect method. The occurrence of AMF in any structure type is noted (out of 25 root intersects). Next, the occurrence of different structures is noted (out of the same 25 root intersects).

|  | AMF | | structure type | | | | |
| --- | --- | --- | --- | --- | --- | --- | --- |
| Sample | NO | YES | hyphae | coil | spore | arbuscules | other |
| 0IW51 | 24 | 1 | 0 | 0 | 0 | 0 | 0 |
| 0IW55 | 25 | 0 | 0 | 0 | 0 | 0 | 0 |
| 0IW71 | 25 | 0 | 0 | 0 | 0 | 0 | 0 |
| 1IW33 | 1 | 24 | 24 | 4 | 10 | 10 | 0 |
| 1IW52 | 4 | 21 | 21 | 2 | 4 | 1 | 1 |
| 1IW15 | 2 | 23 | 23 | 1 | 7 | 2 | 0 |
| 2IW86 | 16 | 9 | 9 | 2 | 8 | 5 | 0 |
| 2IW35 | 8 | 17 | 17 | 2 | 4 | 6 | 0 |
| 2IW46 | 9 | 16 | 16 | 2 | 0 | 3 | 0 |
| 1IW13 | 24 | 1 | 1 | 0 | 0 | 0 | 0 |
| 0SL39 | 25 | 0 | 0 | 0 | 0 | 0 | 0 |
| 0SL14 | 24 | 1 | 1 | 0 | 1 | 0 | 0 |
| 0SL17 | 25 | 0 | 0 | 0 | 0 | 0 | 0 |
| 0DB74 | 25 | 0 | 0 | 0 | 0 | 0 | 0 |
| 0DB86 | 25 | 0 | 0 | 0 | 0 | 0 | 0 |
| 0DB49 | 25 | 0 | 0 | 0 | 0 | 0 | 0 |
| 0MW52 | 25 | 0 | 0 | 0 | 0 | 0 | 0 |
| 0MW86 | 24 | 1 | 0 | 1 | 0 | 0 | 0 |
| 0MW15 | 25 | 0 | 0 | 0 | 0 | 0 | 0 |
| 1DB45 | 25 | 0 | 0 | 0 | 0 | 0 | 0 |
| 1DB34 | 8 | 17 | 15 | 0 | 17 | 0 | 0 |
| 1DB33 | 0 | 25 | 25 | 1 | 24 | 8 | 0 |
| 2DB46 | 13 | 12 | 12 | 1 | 3 | 4 | 0 |
| 2DB11 | 21 | 4 | 4 | 0 | 0 | 0 | 0 |
| 1MW48 | 6 | 19 | 14 | 0 | 8 | 2 | 0 |
| 1MW85 | 24 | 1 | 1 | 0 | 0 | 1 | 0 |
| 1MW81 | 12 | 13 | 13 | 0 | 8 | 4 | 0 |
| 2MW23 | 21 | 4 | 4 | 0 | 0 | 2 | 0 |
| 2MW12 | 13 | 12 | 9 | 1 | 2 | 6 | 0 |
| 2MW81 | 12 | 13 | 13 | 1 | 0 | 10 | 0 |
| 1SL10 | 17 | 8 | 8 | 0 | 0 | 2 | 0 |
| 1SL29 | 18 | 7 | 7 | 0 | 6 | 0 | 0 |
| 1SL37 | 17 | 8 | 8 | 0 | 0 | 1 | 0 |
| 2SL48 | 10 | 15 | 15 | 4 | 0 | 3 | 0 |
| 2SL25 | 9 | 16 | 15 | 0 | 12 | 2 | 0 |
| 2SL49 | 13 | 12 | 11 | 0 | 0 | 9 | 0 |

**Table S5. Summary of Statistical Analyses.**

Statistical summary of models for (A) mycorrhizal colonization, (B) mycorrhizal growth response above- and below-ground for all species together and each species separately, (C) specific root length, (D) correlations between mycorrhizal growth response and specific root length and (E) mycorrhizal growth response and mycorrhizal colonization.

| **A. Mycorrhizal Colonization** | | | | |  |
| --- | --- | --- | --- | --- | --- |
| *All mycorrhizal treatments* | | | | |  |
| Effect | Num DF | Den DF | F-value | P-value | Partial Eta Sq |
| treatment | 2 | 34 | 68.091 | 0.822 | 0.800 |
| *Sterile versus AMF* | | | | |  |
| Effect | Num DF | Den DF | F-value | P-value | Partial Eta Sq |
| treatment | 1 | 34 | 34.070 | <2e-16 | 0.500 |
| *Treatement by Pop* | | | | |  |
| Effect | Num DF | Den DF | F-value | P-value | Partial Eta Sq |
| treatment | 1 | 3 | 0.456 | 0.408 | 0.130 |
| species | 3 | 4 | 2.196 | 0.231 | 0.620 |
| poptype | 1 | 2 | 16.246 | 0.000 | 0.890 |
| poptype*treatment | 1 | 8 | 1.960 | 0.163 | 0.200 |
| **B. Mycorrhizal Growth Response** | | | | |  |
| *Aboveground across species* | | | | |  |
| Effect | Num DF | Den DF | F-value | P-value | Partial Eta Sq |
| lheight_i*species | 4 | 243 | 20.580 | <.0001 | 0.250 |
| treatment | 2 | 176 | 157.090 | <.0001 | 0.640 |
| species | 3 | 240 | 2.790 | 0.041 | 0.030 |
| species*treatment | 6 | 177 | 7.440 | <.0001 | 0.200 |
| poptype | 1 | 22.1 | 0.500 | 0.488 | 0.020 |
| poptype*treatment | 2 | 174 | 2.490 | 0.086 | 0.030 |
| species*poptype | 3 | 21.1 | 0.900 | 0.460 | 0.110 |
| specie*poptyp*treatment | 6 | 174 | 2.380 | 0.031 | 0.080 |
| *Aboveground Dogbane* | | | | |  |
| Effect | Num DF | Den DF | F-value | P-value | Partial Eta Sq |
| lheight_i | 1 | 47 | 10.540 | 0.002 | 0.180 |
| treatment | 2 | 32 | 3.190 | 0.054 | 0.170 |
| poptype | 1 | 19.1 | 0.030 | 0.858 | 0.002 |
| poptype*treatment | 2 | 31.8 | 1.320 | 0.282 | 0.080 |
| *Aboveground Ironweed* | | | | |  |
| Effect | Num DF | Den DF | F-value | P-value | Partial Eta Sq |
| lheight_i | 1 | 76.7 | 18.950 | <.0001 | 0.200 |
| treatment | 2 | 9.59 | 64.780 | <.0001 | 0.930 |
| poptype | 1 | 8.34 | 0.360 | 0.566 | 0.040 |
| poptype*treatment | 2 | 9.34 | 5.700 | 0.024 | 0.550 |
| *Aboveground Milkweed* | | | | |  |
| Effect | Num DF | Den DF | F-value | P-value | Partial Eta Sq |
| lheight_i | 1 | 52.9 | 4.410 | 0.041 | 0.080 |
| treatment | 2 | 35.2 | 68.490 | <.0001 | 0.800 |
| poptype | 1 | 17.2 | 4.900 | 0.041 | 0.220 |
| poptype*treatment | 2 | 35 | 0.090 | 0.912 | 0.005 |
| *Aboveground Solidago* | | | | |  |
| Effect | Num DF | Den DF | F-value | P-value | Partial Eta Sq |
| lheight_i | 1 | 77.6 | 43.000 | <.0001 | 0.360 |
| treatment | 2 | 9.63 | 96.360 | <.0001 | 0.950 |
| poptype | 1 | 4.68 | 0.190 | 0.680 | 0.040 |
| poptype*treatment | 2 | 9.34 | 1.100 | 0.372 | 0.190 |
| *Belowground across species* | | | | |  |
| Effect | Num DF | Den DF | F-value | P-value | Partial Eta Sq |
| lheight_i*species | 4 | 242 | 12.970 | <.0001 | 0.180 |
| treatment | 2 | 179 | 103.180 | <.0001 | 0.540 |
| species | 3 | 233 | 0.390 | 0.761 | 0.005 |
| species*treatment | 6 | 179 | 5.470 | <.0001 | 0.150 |
| poptype | 1 | 18.1 | 0.000 | 0.986 | 0.000 |
| poptype*treatment | 2 | 177 | 0.320 | 0.728 | 0.004 |
| species*poptype | 3 | 17.3 | 0.380 | 0.771 | 0.060 |
| species*poptyp*treatment | 6 | 177 | 2.250 | 0.041 | 0.070 |
| *Belowground Dogbane* | | | | |  |
| Effect | Num DF | Den DF | F-value | P-value | Partial Eta Sq |
| lheight_i | 1 | 46.8 | 2.320 | 0.135 | 0.050 |
| treatment | 2 | 32 | 1.810 | 0.181 | 0.100 |
| poptype | 1 | 3.67 | 0.060 | 0.823 | 0.020 |
| poptype*treatment | 2 | 31.7 | 1.790 | 0.184 | 0.100 |
| *Belowground Ironweed* | | | | |  |
| Effect | Num DF | Den DF | F-value | P-value | Partial Eta Sq |
| lheight_i | 1 | 83 | 8.560 | 0.004 | 0.090 |
| treatment | 2 | 83 | 32.740 | <.0001 | 0.440 |
| poptype | 1 | 83 | 0.310 | 0.580 | 0.004 |
| poptype*treatment | 2 | 83 | 3.310 | 0.041 | 0.070 |
| *Belowround Milkweed* | | | | |  |
| Effect | Num DF | Den DF | F-value | P-value | Partial Eta Sq |
| lheight_i | 1 | 48.4 | 3.430 | 0.070 | 0.070 |
| treatment | 2 | 5.37 | 67.350 | 0.000 | 0.960 |
| poptype | 1 | 2.55 | 0.510 | 0.535 | 0.170 |
| poptype*treatment | 2 | 5.29 | 0.390 | 0.697 | 0.130 |
| *Belowground Solidago* | | | | |  |
| Effect | Num DF | Den DF | F-value | P-value | Partial Eta Sq |
| lheight_i | 1 | 79.5 | 34.350 | <.0001 | 0.300 |
| treatment | 2 | 9.7 | 63.180 | <.0001 | 0.930 |
| poptype | 1 | 4.82 | 0.310 | 0.605 | 0.060 |
| poptype*treatment | 2 | 9.45 | 0.330 | 0.725 | 0.070 |
| **C. Specific Root Length** | | | | | |
| *Species* | | | | | |
| Effect | Num DF | Den DF | F-value | P-value | Partial Eta Sq |
| species | 3 | 4.5587 | 3.935 | 0.096 | 0.720 |
| *Species by Population* | | | | | |
| Effect | Num DF | Den DF | F-value | P-value | Partial Eta Sq |
| species | 3 | 84 | 0.315 | 0.815 | 0.010 |
| poptype | 1 | 84 | 0.005 | 0.946 | 0.000 |
| species:poptype | 3 | 84 | 0.077 | 0.973 | 0.003 |
| **D. Mycorrhizal Growth Response and Specific Root Length** | | | | | |
| Effect | Num DF | Den DF | F-value | P-value | Partial Eta Sq |
| RL.mass | 1 | 17 | 0.535 | 0.475 | 0.030 |
| species | 3 | 17 | 0.821 | 0.500 | 0.130 |
| RL.mass:species | 3 | 17 | 5.123 | 0.010 | 0.470 |
| **E. Mycorrhizal Growth Response and Mycorrhizal Colonization** | | | | | |
| Effect | Num DF | Den DF | F-value | P-value | Partial Eta Sq |
| lt.propcol | 1 | 3.9405 | 60.651 | 0.002 | 0.940 |
| poptype | 1 | 2.9475 | 8.495 | 0.063 | 0.740 |
| species | 3 | 2.8044 | 10.013 | 0.051 | 0.910 |
| lt.propcol:poptype | 1 | 5.9995 | 14.395 | 0.009 | 0.710 |
| lt.propcol:species | 3 | 4.5675 | 8.981 | 0.023 | 0.860 |

**Table S5. Variance components**

Variance components models for (A) mycorrhizal colonization, (B) mycorrhizal growth response above- and below-ground for all species together and each species separately, (C) specific root length, (D) correlations between mycorrhizal growth response and specific root length and (E) mycorrhizal growth response and mycorrhizal colonization.

| **A. Mycorrhizal Colonization** | | | | |
| --- | --- | --- | --- | --- |
| *All mycorrhizal treatments* | | | | |
| Random effect | Variance | Std.Dev. |  |  |
| Species:Pop | 0.5417 | 0.736 |  |  |
| *Sterile versus AMF* | | | | |
| Random effect | Variance | Std.Dev. |  |  |
| Species:Pop | 0.5512 | 0.7424 |  |  |
| *Treatement by Pop* | | | | |
| Random effect | Variance | Std.Dev. |  |  |
| Species:Pop:Treatment | 1.36E-01 | 0.36928 |  |  |
| Species:Pop | 1.44E-07 | 0.000379 |  |  |
| **B. Mycorrhizal Growth Response** | | | | |
| *Aboveground across species* | | | | |
|  | Estimate | Standard Error | Z Value | Pr > Z |
| spec*popt*popr*treat | 0 | . | . | . |
| specie*poptyp*poprep | 3.1983 | 7.9396 | 0.4 | 0.3435 |
| spec*popt*popr*speci | 30.0711 | 14.4563 | 2.08 | 0.0188 |
| Residual | 148.47 | 15.9953 | 9.28 | <.0001 |
| *Aboveground Dogbane* | | | | |
|  | Estimate | Standard Error | Z Value | Pr > Z |
| poptype*poprep | 0 | . | . | . |
| poptyp*poprep*treatm | 0 | . | . | . |
| poptyp*poprep*specie | 62.7563 | 40.5131 | 1.55 | 0.0607 |
| Residual | 140.01 | 35.2799 | 3.97 | <.0001 |
| *Aboveground Ironweed* | | | | |
|  | Estimate | Standard Error | Z Value | Pr > Z |
| poptype*poprep | 6.6622 | 19.2837 | 0.35 | 0.3649 |
| poptyp*poprep*treatm | 1.3863 | 26.4065 | 0.05 | 0.4791 |
| poptyp*poprep*specie | 18.5174 | 32.9421 | 0.56 | 0.287 |
| Residual | 215.09 | 45.9031 | 4.69 | <.0001 |
| *Aboveground Milkweed* | | | | |
|  | Estimate | Standard Error | Z Value | Pr > Z |
| poptype*poprep | 0 | . | . | . |
| poptyp*poprep*treatm | 0 | . | . | . |
| poptyp*poprep*specie | 15.7945 | 16.2987 | 0.97 | 0.1663 |
| Residual | 82.7422 | 19.8212 | 4.17 | <.0001 |
| *Aboveground Solidago* | | | | |
|  | Estimate | Standard Error | Z Value | Pr > Z |
| poptype*poprep | 15.6006 | 21.1185 | 0.74 | 0.23 |
| poptyp*poprep*treatm | 3.4871 | 16.3371 | 0.21 | 0.4155 |
| poptyp*poprep*specie | 19.9855 | 20.8575 | 0.96 | 0.169 |
| Residual | 126.17 | 26.9574 | 4.68 | <.0001 |
| *Belowground across species* | | | | |
|  | Estimate | Standard Error | Z Value | Pr > Z |
| spec*popt*popr*treat | 0 | . | . | . |
| specie*poptyp*poprep | 7.5208 | 11.7313 | 0.64 | 0.2607 |
| spec*popt*popr*speci | 29.7741 | 16.6969 | 1.78 | 0.0373 |
| Residual | 189.03 | 20.2034 | 9.36 | <.0001 |
| *Belowground Dogbane* | | | | |
|  | Estimate | Standard Error | Z Value | Pr > Z |
| poptype*poprep | 2.1326 | 36.1113 | 0.06 | 0.4765 |
| poptyp*poprep*treatm | 0 | . | . | . |
| poptyp*poprep*specie | 82.1834 | 55.2087 | 1.49 | 0.0683 |
| Residual | 162.7 | 41.0094 | 3.97 | <.0001 |
| *Belowground Ironweed* | | | | |
|  | Estimate | Standard Error | Z Value | Pr > Z |
| poptype*poprep | 0 | . | . | . |
| poptyp*poprep*treatm | 0 | . | . | . |
| poptyp*poprep*specie | 0 | . | . | . |
| Residual | 329.09 | 51.0842 | 6.44 | <.0001 |
| *Belowround Milkweed* | | | | |
|  | Estimate | Standard Error | Z Value | Pr > Z |
| poptype*poprep | 4.186 | 18.2456 | 0.23 | 0.4093 |
| poptyp*poprep*treatm | 5.7258 | 13.8197 | 0.41 | 0.3393 |
| poptyp*poprep*specie | 32.3898 | 20.3062 | 1.6 | 0.0553 |
| Residual | 63.3802 | 16.6268 | 3.81 | <.0001 |
| *Belowground Solidago* | | | | |
|  | Estimate | Standard Error | Z Value | Pr > Z |
| poptype*poprep | 25.5881 | 30.5875 | 0.84 | 0.2014 |
| poptyp*poprep*treatm | 13.6222 | 21.3361 | 0.64 | 0.2616 |
| poptyp*poprep*specie | 26.8963 | 22.6157 | 1.19 | 0.1172 |
| Residual | 127.73 | 27.4699 | 4.65 | <.0001 |
| **C. Specific Root Length** | | | | |
| *Species* | | | | |
|  | Variance | Std.Dev. |  |  |
| poptype:species | 0.005799 | 0.07615 |  |  |
| Residual | 0.062392 | 0.24978 |  |  |
| *Species by Population* | | | | |
|  | Variance | Std.Dev. |  |  |
| poptype:species | 0.14591 | 0.382 |  |  |
| Residual | 0.06284 | 0.2507 |  |  |
|  |  |  |  |  |
| **D. Mycorrhizal Growth Response and Specific Root Length** | | | | |
|  | Variance | Std.Dev. |  |  |
| poptype:species | 0 | 0 |  |  |
| Residual | 0.1529 | 0.3911 |  |  |
|  |  |  |  |  |
| **E. Mycorrhizal Growth Response and Mycorrhizal Colonization** | | | | |
|  | Variance | Std.Dev. |  |  |
| species:poptype | 0.17134 | 0.4139 |  |  |
| Residual | 0.01187 | 0.1089 |  |  |

**Figure S1. Mycorrhizal colonization across study treatments.** GLM shows that mycorrhizal treatments showed significantly higher colonization than sterile treatments (p < 0.001).

**
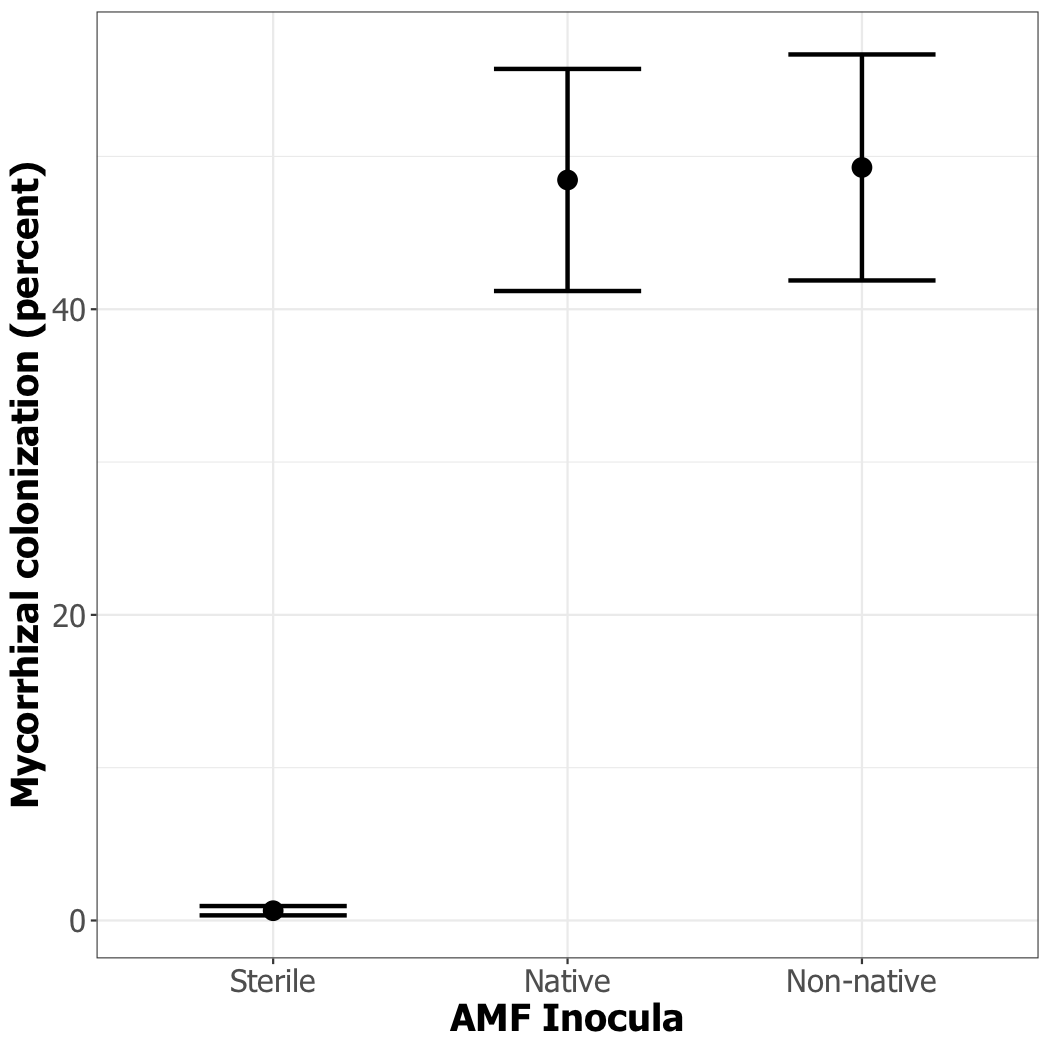
**

**Figure S2. Root length differences across study species.** Linear model shows that there is a marginally significant difference in root length between species, with Dogbane showing longer SRL compared Ironweed (p = 0.1).

**Figure S3. Species differences in relationship between mycorrhizal colonization and response.** The relationship between logit colonization proportion and mycorrhizal growth response depends on species, with Ironweed showing a more positive relationship (p = 0.03).


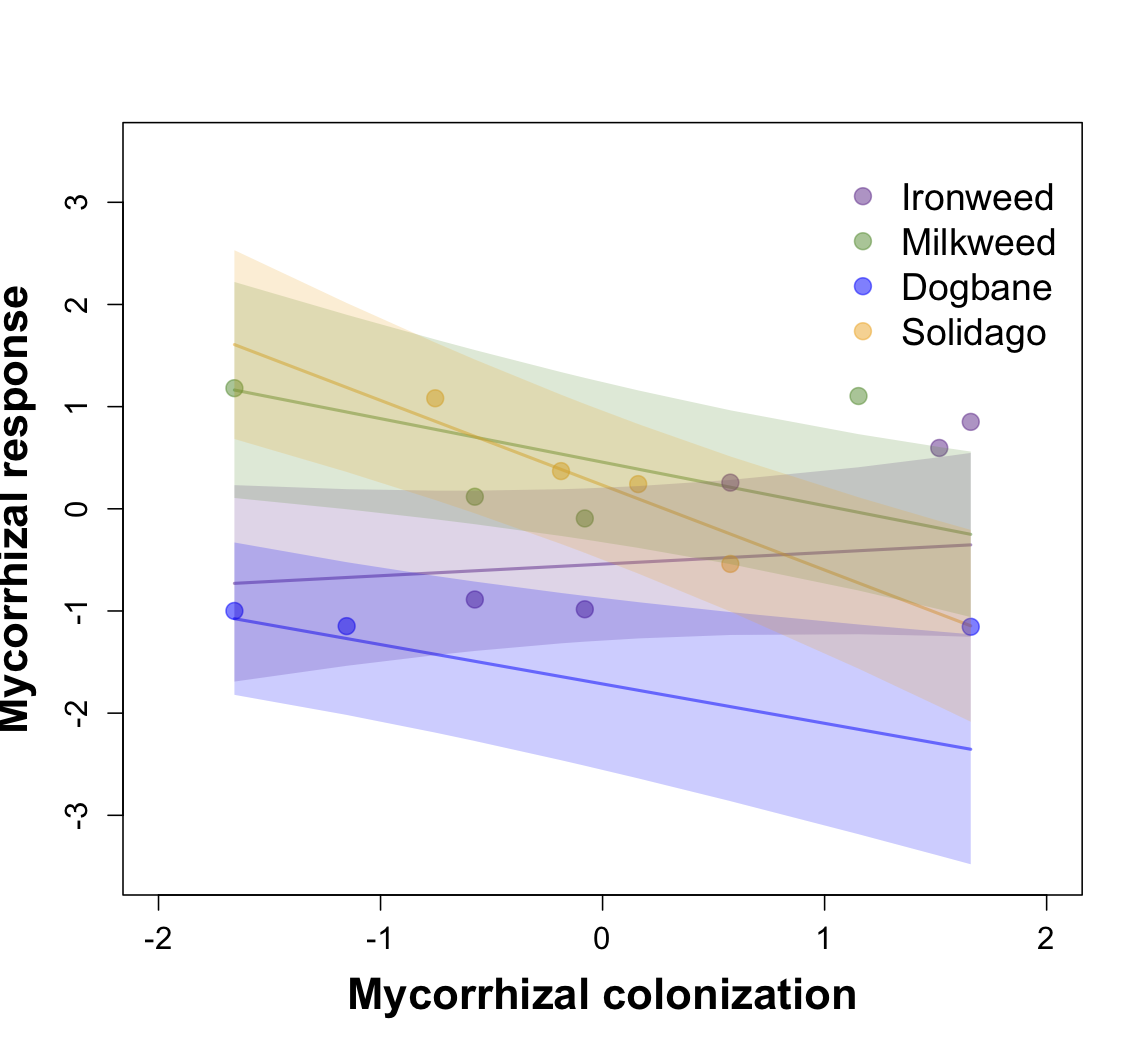

Supplement: Supplementary file 1 — Appendix S1 Supporting Information [file ECE3-12-e9097-s001.docx]
